# Aging Effects on a Driver Position Sensor Integrated into a Woven Fabric †

**DOI:** 10.3390/s25123797

**Published:** 2025-06-18

**Authors:** Marc Martínez-Estrada, Ignacio Gil, Raúl Fernández-García

**Affiliations:** Departament of Electronic Engineering, Universitat Politecnica de Catalunya, ESEIAAT, Colom 1, 08222 Terrassa, Spain; ignasi.gil@upc.edu (I.G.); raul.fernandez-garcia@upc.edu (R.F.-G.)

**Keywords:** presence sensor, woven, textile, aging, reliability, capacitance

## Abstract

A textile woven presence sensor was previously presented to demonstrate its functionality in eliminating some false positives on car seat presence sensors. After studying the functionality, the next characteristic that the textile sensor should demonstrate is its reliability. The woven sensor was prepared to be tested against ageing. The ageing cycle was prepared according to the UNE-EN ISO 17228:2015 standard. Nine woven sensors are prepared, seven of them face the aging test, and two are selected as reference sensors. The characterization of the woven sensor has been carried out through a microcontroller measurement circuit that obtains the cycles to charge the sensor. Comparison of the results obtained shows that the effects of ageing are negligible. The behavior of the aged sensors is similar to that of the reference sensors, indicating that the variations in the values of both aged and reference sensors are provoked by the environmental conditions during the measurements. To support this argument, a statistical study based on a t-Student analysis was carried out. After 4 ageing cycles, the functionality of the sensors remains unaffected. This research proves the reliability of the woven textile sensor, which encourages its use in automotive applications.

## 1. Introduction

In recent years, researchers have been working to provide alternatives to standard electronic devices by replacing them with new technologies using flexible solutions. The use of highly foldable and flexible materials has enabled researchers to apply new technologies in the most demanding fields, such as biomedicine [1] and health monitoring [2]. Merces et al. presented dynamically morphing microelectronics manufactured with highly foldable materials. These materials can be used to create soft foldable supercapacitors and bio-adaptive devices with a dynamic shape, enabling smart implant technologies. The results presented by Merces et al. demonstrate the advantages of applying these foldable and flexible properties to the development of new electronic devices. One field that could benefit significantly from flexible technologies is electronic sensors. Flexible sensors have been presented as an alternative to standard sensors to improve their applicability and overcome the limitations of conventional electronic sensors. In particular, presence and pressure sensors for automotive applications, such as car seats or gesture monitoring, have been developed [3,4].

Presence and pressure sensors have been used for years to provide a reliable system for confirming seatbelt use. The unbuckled alarm is activated the moment it detects the driver over the seat and the seatbelt is not fastened. Occupancy information has traditionally been collected using a resistive sensor, a Force-Sensitive Resistor (FSR). FSR sensors detect occupancy by the change in pressure on the seat surface, which changes the resistance of the sensor. To be effective on the vehicle, the sensor system must have a minimum pressure to be activated, which is directly dependent on the weight experienced by the seat surface. This fact leaves situations where a false positive could be detected, such as a heavy object or bag that exceeds the minimum pressure of activation. In recent years, researchers have worked to provide alternatives to the standard sensors by replacing them with new technologies. This is the case for textile or flexible sensors. The substitution could be based on the need to improve the sensor characteristics for a specific application or the flexibility of integration. A possible substitution is the flexible presence and pressure sensor for applications such as car seats or gesture monitoring.

Researchers have been working on flexible sensors that can detect pressure or position, with the aim of replacing sensors such as FSR sensors [5,6,7,8,9,10,11]. The advantages of flexible sensors, such as the ease of installation on a variety of surfaces and substrates, provide compelling arguments for them to be considered a proper substitute. However, as the operating principle of these flexible pressure sensors is the same as that of FSRs, the possibility of having the same false positives in occupancy detection is still an issue to be resolved. The false positives come from the fact that the variation in pressure across the sensing zone does not mean that a person is sitting.

Focusing on solving the false positives issue. A capacitive textile presence sensor has been presented [12,13,14], but it has also been presented in previous works by the authors [15,16]. The capacitive textile sensor has proven its functionality as a driver detector and as a driver position measure when some sensors are placed in an array structure over the seat and the back seat. The previous work [15] demonstrated the ability of the sensor to discriminate between a person occupying the seat, a bag pack occupying the seat, and nothing over the sensor, which could solve the false positive problem on FSR. This ability relies on the working principle of the capacitive sensor, which is based on changes in the surrounding permittivity. When a person sits over the sensor, the permittivity in close contact with the sensor increases drastically due to the body’s high permittivity, causing a significant increase in the sensor value. However, when a bag comes into contact with the sensor, the sensor value does not increase significantly, as it is unable to differentiate between the two. Additionally, the use of a capacitive woven sensor on the seat and the back seat proved that the driving position could be monitored to prevent dangerous situations caused by fatigue or health problems such as heart attack.

The capacitive woven sensor, which is integrated into the car seat’s upholstery, is easy to install and replace. It can also be integrated into every single seat in a car, bus, or other vehicle because it is low-cost. However, the capacitive woven sensor has to prove its reliability. The authors prove the pilling resistance of the capacitive woven sensor by performing a Martindale test on the woven fabric with integrated sensors [17]. The sensors have passed the first durability and reliability test against pilling, but now have to pass another reliability test against ageing effects.

The effects of ageing have been studied on electronic and textile sensors to ensure their functionality and durability throughout their entire life cycle [18,19,20,21]. The characteristics of the ageing test could be defined by the integration substrate, the measured parameter, or the construction materials. Overall, tension, temperature, and humidity are the most common variables to be changed in ageing tests.

The textile sensor must guarantee its reliability throughout its life cycle in order to maintain its usability [22,23]. For this reason, integrated sensors on textiles must meet the same requirements as the fabric in which they are integrated. Their functionality, measurement, and position data have been studied before and after the ageing cycle. The researchers aim to demonstrate the reliability of the capacitive woven sensor for the automotive industry, but also for other industries such as healthcare and ergonomics.

The paper presented is an extension of the work presented at the Eurosensors 2023 Conference, in Lecce, Italy [16]. The present research seeks to extend the previously obtained results by evaluating the effect of ageing on the capacitive woven sensor.

## 2. Materials and Methods

**Figure 1:**
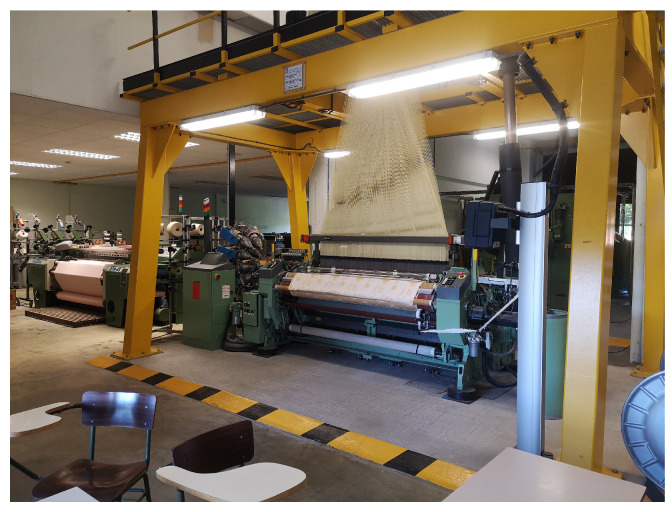
Woven process due on a Jacquard Stäubli LX1600B.

The capacitive woven sensor tested consists of a woven fabric made with conventional textile yarns and conductive yarns. The conductive yarns are used to form an interdigital structure within the woven structure using a Stäubli LX1600B Jacquard machine, by Stäubli, Horgen (Switzerland) as shown in Figure 1.

**Figure 2:**
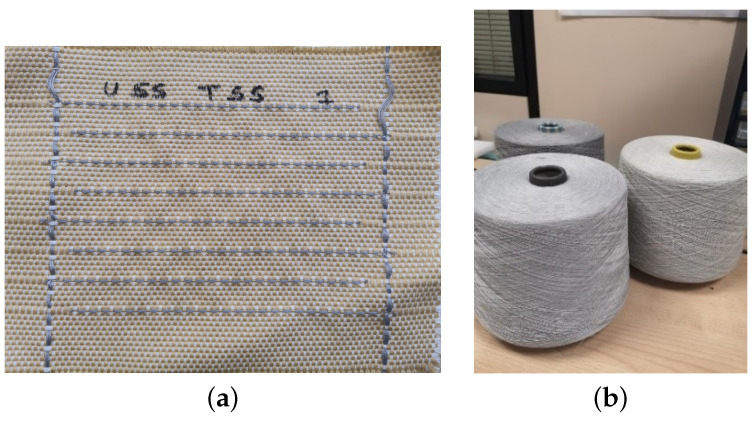
Woven sensor presented on the Eurosensors 2023 [16]. (**a**) Woven capacitive sensor (10 × 11 cm); (**b**) conductive stainless steel yarn.

The fabric is made with cotton yarns in the warp and cotton/polyester mixed yarn in the weft. During the manufacturing process, conductive yarns are introduced in the weft and warp directions. Previous work by the authors explains the process in detail [15]. Figure 2a shows the capacitive woven sensor presented.

The conductive yarn selected for the manufacture of the woven sensor is a commercially available conductive yarn from Bekaert, Zwevegem (Belgium). The yarn was manufactured using the ring yarn method, in which the conductive stainless steel fibres were mixed with polyester fibres in a ratio of 40/60%. The methodology used results in a highly resistant yarn with the feel of a common textile manufacturing yarn, as shown in Figure 2b. Nine sensors are manufactured for the study. Two of these are used as reference sensors, which means they are not exposed to the ageing test.

**Figure 3:**
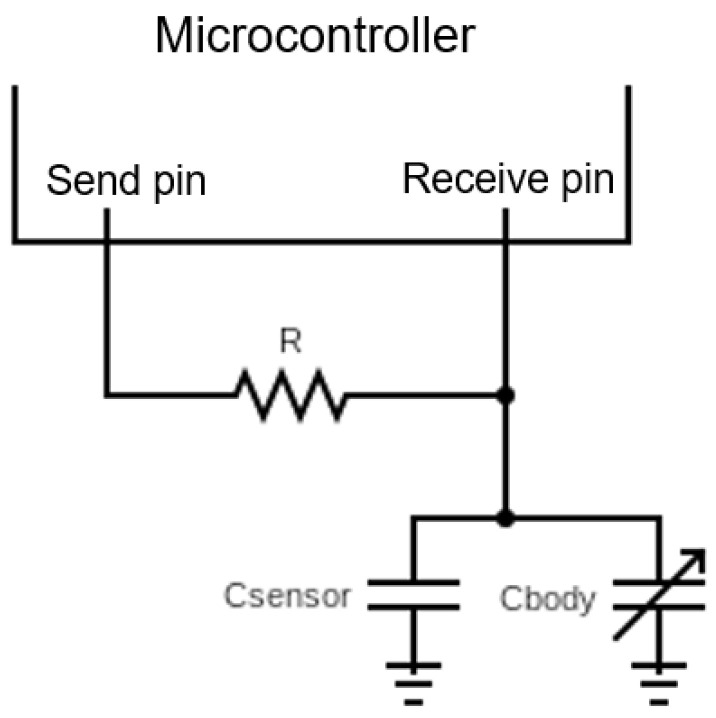
Measurement microcontroller cycle count circuit.

The sensor measurement is performed using a microcontroller from the ATSAMD21 family. The circuit is shown in Figure 3. On the circuit, two pins of the microcontroller are connected to each other by a resistor, which they are called send and receive pin. On the *receive pin* side, one of the electrodes of the capacitive sensor is connected. The other electrode of the sensor is connected to ground, and it must be taken into account that the body works as a parallel capacitor with the woven sensor. The microcontroller carries out a procedure in which it counts the clock cycles needed for the sensor capacitor to be fully charged. The *send pin* is set to high state, and the current flowing through the resistor then starts to charge the sensor capacitor. The microcontroller starts counting the clock cycles the moment it changes the voltage on the *receive pin* for the first time. When the *receive pin* reaches the same voltage value as the *send pin*, the cycle count value is taken as it means that the capacitor is fully charged. Due to the stability of the values obtained by the circuit presented, this measurement method would be chosen for a future application of the woven presence sensor.

The effects of ageing on a product can only be observed after years of use. For some studies, it is not feasible to wait for several years to obtain the reliability of a product, so accelerated ageing tests become relevant to obtain an estimation. In the case of the woven sensor presented, a regulation related to car upholstery is used to evaluate the reliability [24]. In the UNE-EN ISO 17228:2015 standard [24], the conditions for an accelerated ageing test are defined for different cases. The conditions related to the car upholstery were as follows: first, a cycle of 24 ± 1 h at a temperature of 50 ± 2 °C and a relative humidity (RH) of 90 ± 5%, followed by 24 h in a normalized atmosphere (23 °C and 50% RH). A CCK-25/48 Dycometal climatic chamber by Dycometal Viladecans (Viladecans, Spain) was used to carry out the study. The seven woven sensors were placed in the climatic chamber to face the conditions of 50 ± 2 °C and 90 ± 5% RH for 24 h. The woven sensors were then removed from the climate chamber and exposed to a normalized atmosphere for 24 h. The cycle was repeated 4 times for each sensor.

At the end of each ageing cycle, the sensor was measured using the method described above, with a microcontroller that has not undergone the ageing cycle. The capacitive values obtained were divided into *rest values*, which are the values obtained by the microcontroller when the sensor is not occupied, *initial values*, which are the presence values obtained prior to the accelerated ageing test, and *aging test values*, which correspond to the presence values obtained after each accelerated ageing cycle.

## 3. Results and Discussion

In accordance with the presence woven sensor research presented during the Eurosensor 2023 [16], the response of the sensor to different driver positions is shown. The driver is a male, 32 years old, 77 kg, and 1.76 m tall.

**Figure 4:**
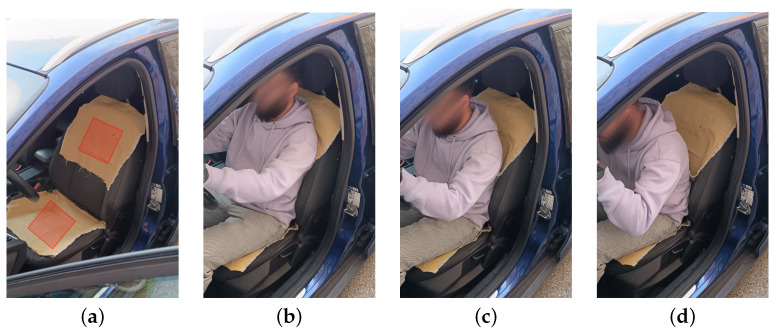
Definition of the different driver states measured by the driver position sensor system. (**a**) State 0: Void, no person, Red box indicates the place where the sensor is integrated; (**b**) State 1: Normal position; (**c**) State 2: Back slightly separated; (**d**) State 3: Back separated.

Figure 4 shows the different states that the sensor could detect during driver monitoring. There are four important states. State 0 reflects when the car seat is empty. States 1, 2, and 3 reflect three different states regarding the backrest position during the driving period.

**Figure 5:**
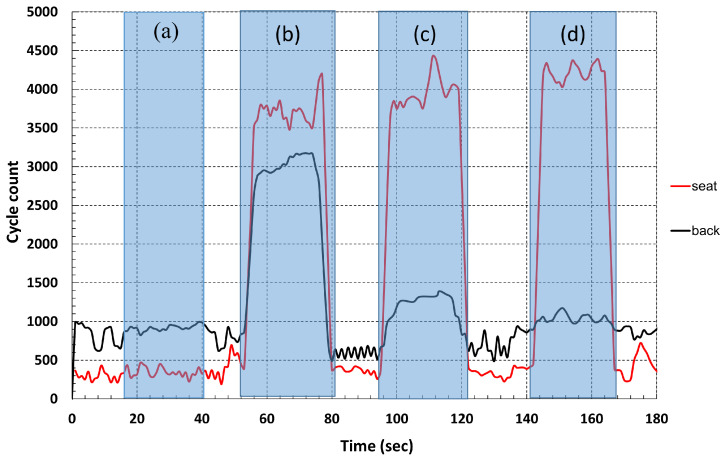
Response measured by the woven sensors integrated on the car seat. The measurement method used is the cycle count measurement circuit. (**a**) State 0; (**b**) State 1; (**c**) State 2; (**d**) State 3.

The response of the sensor during these four states, measured by the cycle count measurement circuit, is shown in Figure 5. In the graph, the data obtained from the woven sensor placed on the backrest (black line) and the seat (red line) of the car seat provide information to identify different positions and situations of the driver. At rest (State 0), there is no person sitting above the sensor (Figure 4a), which is shown by the constant values of the cycle count measured by the microcontroller. For the next positions taken by the driver over the car seat, shown in Figure 4b–d, the graph shows how the sensor response is related to the position taken by the driver, States 1, 2, and 3, maintaining the value for the seat sensor and reducing the cycle count value for the backrest sensor as the distance between the driver’s back and the seat increases.

At this point, as the sensor response demonstrates its functionality, it is important to evaluate the reliability of the sensor. A total of nine sensors are manufactured and prepared for the ageing tests. Of these, two sensors are designated as reference sensors (SR1 and SR2), meaning that they are not subjected to the ageing cycles in the climate chamber. However, these reference sensors are measured alongside the aged sensors (S1–S7) at each stage and after each ageing cycle. This procedure ensures that any environmental changes during the test period are taken into account, thus maintaining consistent and reliable comparisons.

**Figure 6:**
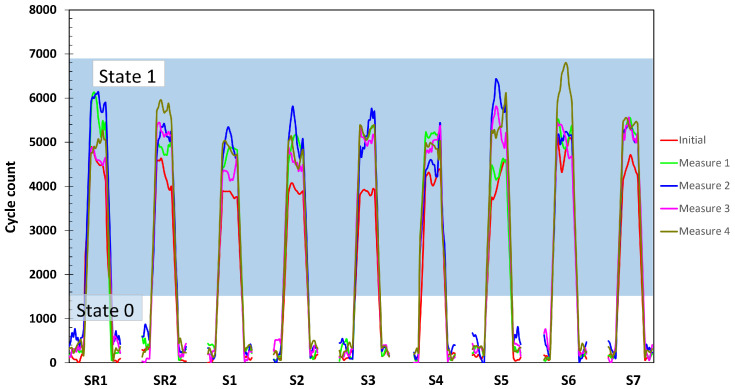
Study of the individual response of presence woven sensors after the aging cycles by the cycle count measurement method. Shadowed zone represents the woven reference sensors.

The individual response of the woven sensors before and after each ageing cycle is shown in Figure 6. The measured values represent the cycle count change from State 0 (rest values) to State 1 (occupied values), corresponding to the change when presence is detected, and the response to State 1 (Figure 4b). The data are obtained by the cycle count measurement circuit, which is the system designed for real-world applications. This measuring unit enables the system to focus on value variations and improves the ease with which the sensors can be measured. The rest values of the sensors, corresponding to the measured empty seat state, are kept below 800 cycles for all the sensors studied and after each of the ageing cycles. The initial response to State 1, or no ageing cycle (red line), shows a lower response to presence detection than the cycle measurements after the ageing cycles. The difference between the measurements is also observed for the reference woven sensors. After the first ageing cycle, the cycle count values show an increase for all the sensors studied, including the reference sensors. When analyzing the behavior between the ageing sensors and the reference sensors, it is difficult to attribute it to the ageing cycles, as there is no clear trend in the measured values. To further investigate the possible causes and to clarify the effects on the response of the woven sensors, a study of the woven sensor population is carried out for each ageing cycle.

**Figure 7:**
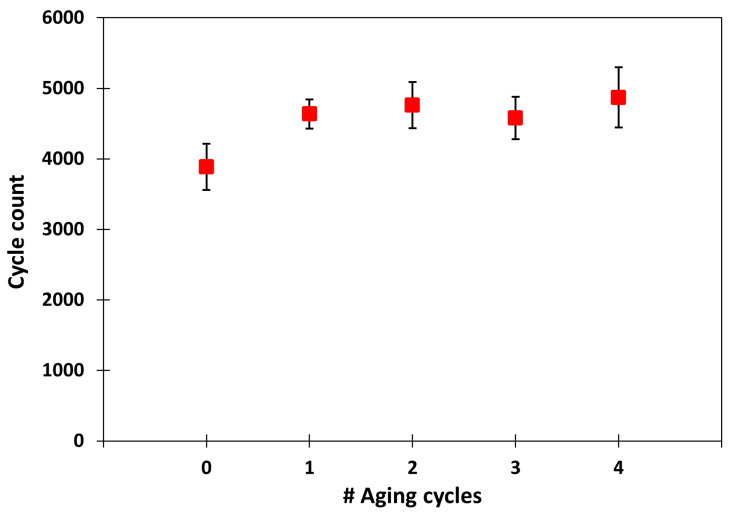
Sample population study of the response of presence woven sensors after the aging cycles.

The cycle count values measured are examined below by grouping the response of all the sample woven fabric sensors. Figure 7 shows the average cycle count values obtained before and after the accelerated ageing cycles. The population averages are obtained by averaging the individual average of each sensor for each ageing condition. The x-axis shows the number of ageing cycles accumulated by the sensors. The error bars represent the standard deviation for each sample population. The cycle count averages are in the range of 3800–4800 cycles. The lowest cycle count value is observed before ageing and takes the value of 3887. After the first ageing cycle and subsequent cycles the values are in the range of 4570–4870 cycle values. The maximum cycle count values are observed after the second and fourth ageing cycles with cycle counts of 4759 and 4870, respectively. The maximum values of the standard deviation are located at the initial state and at the fourth ageing cycle, both around 8.5% of the measured value. As can be seen, the average number of cycles increases and decreases consecutively without any apparent pattern. Similar behavior is observed for the standard deviation values, where no successive increase or decrease is evident with an increase in the number of ageing cycles. The study of the average and standard deviation values of the cycle count after accelerated ageing cycles does not indicate any discernible effect on the behavior of the woven sensor.

Following the analysis of the sample woven sensor values, further statistical analysis is carried out. In this research, t-Student analyses have been conducted to accept or deny that the accelerated ageing test has an identifiable effect on the sensors by determining if the woven sensors tested against ageing still belong to the population of reference sensors. Table 1 presents the data used to evaluate and perform the t-Student analysis.

The table shows the average cycle count of the two reference sensors (${\bar{x}}_{ref}$). Although no accelerated ageing cycles are applied to these sensors, they are measured after each ageing cycle applied to the rest of the sensors to avoid any additional effects.

The next term presented corresponds to the average of the values of the seven sample sensors after each ageing cycle (${\bar{x}}_{sample}$), presented with its standard deviation ($\sigma_{sample}$). The three values presented provide enough information to obtain the calculated t-Student value ($|t_{calc}|$) for each population in the study [25]. The parameter $t_{calc}$ allows one to obtain the t-Student parameter of the actual sample, and it is calculated by the following:

(1) $$t_{calc}=\frac{\bar{x}-\mu }{\frac{s}{\sqrt{n}}}$$

where $\bar{x}$ is the sample mean, μ is the population mean, *s* is the standard deviation, and *n* is the size of the sample. To evaluate the hypothesis of membership for the seven samples, it is necessary to evaluate $|t_{crit}|$. The t-Student for a 95% confidence level (2 sides) with 6 degrees of freedom (df = 7 − 1) is obtained from the t-Student table distribution as 2.447 [25].

Once both *t*-values are obtained, they are compared. As can be seen in all cases, $|t_{calc}\left|\;<\;\right|t_{crit}|$. The result confirms that the samples obtained after each ageing cycle belong to the same population as the values of the reference woven sensor. Another detail that can be observed in Table 1 is based on the comparison between the two average values. Both average values, ${\bar{x}}_{ref}$ and ${\bar{x}}_{sample}$, follow the same increase and decrease behavior between ageing cycles, which means that the overall behavior for reference sensors and sample woven sensors is very similar. The difference between the two averages is also maintained along the study, with a cycle count of around 150. The statistical study carried out with the t-Student comparison of values does not indicate any effect regarding the accelerated ageing cycles done to the seven woven sensors compared to the reference sensors.

**Figure 8:**
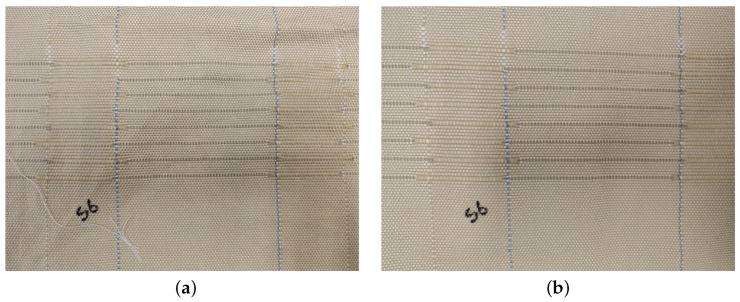
Woven sensor before and after the exposition to all the ageing cycles. (**a**) Sensor before aging cycles; (**b**) sensor after aging cycles.

To conclude the analysis of the ageing effects, Figure 8 shows a picture of the sensor before and after the ageing cycles. As can be seen in the image, there is no appreciable difference in appearance. The texture and shape of the fabric have also been observed and compared with the reference sensor, and any differences detected by the authors can be related with ageing cycles.

Taking into account the analysis of the cycle count values with the statistical study, there is no evidence of any effect on the functionality of the woven presence sensor due to ageing. The research presented demonstrates the reliability of the sensor against ageing effects in an application such as a car seat, where it can be a replacement for conventional FSR presence sensors.

## 4. Conclusions

In this paper, the researchers have presented the results of the reliability of the woven capacitive sensor against ageing cycles, to complement the results of the functionality obtained in previous works by the authors. The conditions set for the test were fixed at 24 ± 1 h at 50 ± 2 °C and 90 ± 5% relative humidity (RH), followed by 24 h in a normalized atmosphere (23 °C and 50% RH). The sensor measurements show different values of the number of cycles, the variations of which are not related to the ageing cycles that the reference sensor also undergoes. A statistical study was carried out, specifically a t-Student comparison. The results of the comparison carried out using the equation $|t_{calc}\left|<\right|t_{crit}|$ indicate that, although the seven woven sensors have undergone four accelerated ageing cycles, they still belong to the same population as the reference sensors. Both conclusions allow researchers to accept that accelerated ageing has no effect on the woven presence sensor, demonstrating its reliability. Finally, the functionality and resistance to pilling demonstrated in previous work by the authors [16,17] is complemented by the reliability demonstrated in the present research, providing sufficient arguments for the claims that the woven presence sensor is a viable replacement for the FSR presence sensor used on car seats and that it more reliably avoids presence detection by objects.

## Figures and Tables

**Table 1:** Statistical study from the measured cycle count values obtained for each accelerated aging cycle.

Measurement Cycle	${\bar{x}}_{\mathrm{ref}}$	$\sigma_{\mathrm{ref}}$	Aging Cycles	${\bar{x}}_{\mathrm{sample}}$	$\sigma_{\mathrm{sample}}$	$|t_{\mathrm{calc}}|$	$|t_{\mathrm{crit}(95\%)}|$	Pertain
0	4062.64	±92.86	0	3887.63	±328.81	1.40	2.447	yes
1	4806.05	±283.20	1	4636.30	±206.42	2.17	2.447	yes
2	4995.24	±258.20	2	4759.67	±327.25	1.90	2.447	yes
3	4733.42	±76.46	3	4579.19	±301.06	1.35	2.447	yes
4	5074.10	±167.38	4	4870.41	±427.13	1.26	2.447	yes

## Data Availability

Data are contained within the article.
